# ApoB/apoA1 is an effective predictor of coronary heart disease risk in overweight and obesity^☆^

**DOI:** 10.1016/S1674-8301(11)60036-5

**Published:** 2011-07

**Authors:** Min Lu, Qun Lu, Yong Zhang, Gang Tian

**Affiliations:** Department of Cardiology, the First Affiliated Hospital of Medical College, Xi'an Jiaotong University, Xi'an, Shaanxi 710061, China.

**Keywords:** apoB/apoA1 ratio, coronary heart disease, overweight, obesity

## Abstract

We investigated the relationship of apoB/apoA1 ratio and coronary heart disease (CHD) in persons who were overweight or obese. The subjects were divided by the body mass indexes (BMI) into the normal weight group (*n*=397, BMI<24 kg/m^2^) and the overweight group (*n*=400, BMI>24 kg/m^2^). Our results showed that the over-weight group had higher blood pressure [(130.15±19.01) mmHg *vs* (123.66±18.70) mmHg] and higher levels of blood sugar [(7.09±2.89) mmol/L *vs* (6.21±2.59) mmol/L], triglyceride [(1.93±1.19) mmol/L *vs* (1.44±0.85) mmol/L], total cholesterol [(4.26±1.06) mmol/L *vs* (4.09±0.99) mmol/L], low-density lipoprotein cholesterol (LDL-C) [(2.56±0.75) mmol/L *vs* (2.39±0.72) mmol/L], and apoB [(0.83±0.27) mg/L *vs* (0.78±0.23) mg/L], and a higher apoB/apoA1 ratio (0.83±0.27 *vs* 0.75±0.25) and lower levels high-density lipoprotein cholesterol [(1.10±0.26) mmol/L *vs* (1.21±0.31) mmol/L] and apoA1 [(1.04±0.20) mg/L *vs* (1.08±0.22) mg/L] than those of the normal weight group (all *P* < 0.05). The prevalence of CHD in the over-weight group in the lowest LDL quartile was almost twice greater than that of the highest apoB/apoA1 quartile, compared with the subjects in the lowest apoB/apoA1 quartile. The higher apoB/apoA1 quartile was in agreement with the higher prevalence of CHD. In the overweight and obesity group, the area under ROC curve (AUC) was the highest for apoB/apoA1 (0.655). The cut-off point of apoB/apoA1 for optimal sensitivity and specificity was at 0.80, with a sensitivity of 57.19% and a specificity of 71.72%. In conclusion, apoB and apoA1 were simple clinical indicators, and the apoB/apoA1 ratio was closely related with CHD in overweight and obese patients. The apoB/apoA1 ratio may provide some useful information in the differential diagnosis.

## INTRODUCTION

Although studies of lipids and the degree of coronary atherosclerosis began in the late 1960s, uncertainty remains which lipid measurement best discriminates the degree of coronary heart disease (CHD). Discussions on which is the “most influential” lipid parameter have been particularly unrewarding. Epidemiological and clinical studies have consistently demonstrated that increased low-density lipoprotein cholesterol (LDL-C) is a major atherogenic lipoprotein for developing CHD and is currently recommended as the primary target for lipid-lowering therapy for the prevention and treatment of cardiovascular disease[1],[2], but many individuals with CHD have normal or even low LDL-C concentrations[3]. Apolipoproteins are important components of lipoprotein particles, and there is accumulating evidence that the measurement of various forms of apolipoproteins may improve the prediction of the risk of cardiovascular disease[4]. Apolipoprotein B (apoB) presents as a single molecule in all potentially atherogenic lipoprotein particles, i.e. very low-density lipoprotein (VLDL), intermediate-density lipoprotein (IDL), and LDL[5]. Thus, a plasma value of total apoB reflects the number of cholesterol and, to some degree, triglyceride-containing particles[6],[7]. Apolipoprotein A1 (apoA1) is the major apolipoprotein associated with high-density lipoprotein (HDL), and a main initiator and “driver of the reverse cholesterol transport”[8],[9]. ApoA1 can also manifest anti-oxidant and anti-inflammatory effects, and it can stimulate both endothelial production of nitric oxide as well as release of prostacyclin from the endothelium[10]. Thus, apoA1 manifests several anti-atherogenic effects[11]. An accumulating body of data indicates that the apoB/apoA1 ratio is a powerful marker of risk for future cardiovascular disease[12], but the different relationships between the plasma apoB, apoA1, apoB/apoA1 ratio and the prevalence of CHD in normal- or over-weight and obese groups have not been consistently shown. Therefore, our objectives were to investigate the possible relationship of apoB/apoA1 ratio and CHD in overweight and obese subjects, and to find whether apoB/apoA1 is better than LDL-C for prediction of cardiac risk, and to identify additional insights into the underlying atherothrombotic disease process in these patients.

## MATERIALS AND METHODS

### Patients and control subjects

This study was approved by the Institutional Ethics Committee of the First Affiliated Hospital of Xi'an Jiaotong University, and informed consent was obtained from all participants. Hospitalized patients with CHD who underwent coronary angiography in the Department of Cardiology, the First Affiliated Hospital Xi'an Jiaotong University from August, 2008 to March, 2010 were considered for enrollment in this study. All the coronary angiography procedures were performed because of clinical indications, typically ischemic chest pain or an abnormal finding in such as electrocardiogram (ECG), troponin, myocardial enzyme, or exercise testing. Patients were enrolled in the study if their angiographic results revealed at least one obstruction with an angiographic luminal diameter narrowing of ≥50%. The CHD subjects were compared with individuals drawn from a background population selected from a large population to study and investigate the preventive factors for cardiovascular disease. The individuals without symptomatic cardiovascular disease, or atherosclerosis during 2008-2009, were included and were chosen to represent the general population of approximately the same age as the patients. In addition, individuals with symptomatic cardiovascular disease, or with hypertension, diabetes, acute and chronic heart failure, acute cerebral hemorrhage or stroke, severe liver disease, chronic wasting disease, thyroid dysfunction, and cancer were excluded. Body mass index (BMI) was calculated as weight in kilograms divided by height in square meters. A BMI of 18.5-23.9 kg/m^2^ was considered normal weight, 24.0-27.9 kg/m^2^ was considered overweight, and ≥28.0 kg/m^2^ was considered obese. All subjects were divided into 2 groups according to BMI: the overweight group [*n*=400, 297 males and 103 females, aged from 32 to 81 years, average age (56.72±9.95) years, BMI>24 kg/m^2^] and the normal weight group [*n*=397, 242 males and 155 females, aged from 32 to 80 years, average age (56.00±11.51) years, BMI<24 kg/m^2^]. Further data of the cases are given in ***Table 1***.

### Research methods

Venous blood samples were obtained from each participant after a fasting period of 10-12 h in the next morning after being hospitalized. Total cholesterol (TC) and triglyceride (TG) were measured by enzymatic colorimetric methods, LDL-C was estimated by the Friedewald equation, and HDL-C was carried out by means of the enzymatic method of elimination. apoB, apoA-1, and C-reactive protein (hs-CRP) measurements were carried out by means of the turbidimetric method. The assays were performed in the clinical laboratory of our hospital.

### Statistical analysis

SPSS for Windows 11.5 was used to analyze all statistical data. The characteristics of the subjects were described as mean±SD for normally distributed continuous variables, or median (interquartile range) for non-normally distributed ones. Non-normal data were log-transformed before statistical analysis. Analysis of variance, *t*-tests, or the Mann–Whitney *U*-test was adopted to assess the differences in mean values as needed. Pearson correlation coefficients were constructed to test the relationships between continuous variables. A logistic regression analysis was performed with the overweight subclass phenotype as dependent BMI. Receiver operating characteristics (ROC) curve analysis was performed for TC, TG, HDL-C, HDL-C, apoB, apoA1, and apoB/apoA1 with respect to subclass phenotype. *P* < 0.05 (2 tailed) was considered as statistically significant. Multiple regression analysis was used to explore whether the lipidic, and apolipoproteic variables were independently associated with development of CHD with normal- or over-weight.

## RESULTS

### The clinical characteristics of the study groups

The clinical characteristics of the study group with normal- or over-weight are presented in ***Table 1***. History of smoking was established if the subject reported smoking ever >10 cigarettes/day for at least 5 years. Among the overweight group, there were more men than women and there were more patients in the over-weight group who had higher blood pressure and blood sugar than in the normal weight group. Furthermore, the overweight group showed a higher potent cardiovascular risk factor than the normal weight group, such as diabetes mellitus, arterial hypertension, with the exception of lipoprotein, and the proportion of patients with CHD in the overweight group was significantly higher than that in the normal weight group.

As seen in ***Table 1***, the overweight group had higher blood pressure, and higher levels of blood sugar, TG, TC, LDL-C, hs-CRP and apoB, and a higher apoB/apoA1 ratio, and lower levels of HDL-C and apoA1 than the normal weight group. The proportion of patients with CHD in the overweight group was higher than those in the normal weight group.

**Table 1:** The demographic and clinical characteristics of study participants

Characteristics	Normal weight (*n*=397)	Overweight (*n*=400)	*P*
Men/women (*n/n*)	242/155	297/103	-
Age (years)	56.00±11.51	56.72±9.95	0.351
Family history of premature CAD, *n*(%)	29(7)	37(9)	0.314
Ever smoked [*n*(%)]	156(42)	179(45)	0.113
History of DM [*n*(%)]	36(9)	60(15)	0.011
History of hypertension [*n*(%)]	54(14)	87(22)	-
CHD [*n*(%)]	161(41)	236(66)	-
BMI (kg/m^2^)	21.63±1.67	26.78±2.29	< 0.001
WHR	0.90±0.08	0.94±0.09	< 0.001
SBP (mmHg)	123.66±18.70	130.15±19.01	< 0.001
DBP (mmHg)	77.56±11.40	81.04±11.81	< 0.001
FG (mmol/L)	6.21±2.59	7.09±2.89	< 0.001
TG (mmol/L)	1.44±0.85	1.93±1.19	< 0.001
TC (mmol/L)	4.09±0.99	4.26±1.06	0.020
LDL-C (mmol/L)	2.39±0.72	2.56±0.75	0.001
HDL-C (mmol/L)	1.21±0.31	1.10±0.26	< 0.001
ApoB (mg/L)	0.78±0.23	0.83±0.27	< 0.001
ApoA1 (mg/L)	1.08±0.22	1.04±0.20	0.016
ApoB/ApoA1	0.75±0.25	0.83±0.27	< 0.001
hs-CRP	0.71(0.58∼0.89)	3.2(2.0∼7.9)	0.001

CAD: coronary artery disease; DM: diabettes mellitus;CHD: coronary heart disease; BMI: body mass index; WHR: waist hip ratio; SBP: systolic blood pressure; DBP: diastolic blood pressure; FG: fasting glucose; TG: triglyceride; TC: total cholesterol; LDL-C: low density lipoprotein cholesterol; HDL-C: high density lipoprotein cholesterol; hs-CRP: hypersensitivity C-reactive protein.

### CHD occurrence by apoB/apoA1 quartiles in the normal and overweight groups

According to apoB/apoAl ratio quartile point, the overweight group were divided into 4 groups: quartile 1 (apoB/apoA1, male 0.31-0.65, female 0.20-0.63); quartile 2 (apoB/apoA1, male 0.65-0.83, female 0.63-0.76); quartile 3 (apoB/apoA1, male 0.83-1.02, female 0.76-0.94); quartile 4 (apoB/apoA1, male 1.02-1.96, female 0.94-1.54). As shown in ***Table 2***, in the over-weight group, the prevalence of CHD increased by 9% from apoB/apoA1 ratio quartile 1 to quartile 3 and by 14% from quartile 3 to quartile 4. Quartile 2 and quartile 3 had no significant difference. A clear increase of TG, TC, LDL-C, and apoB levels was observed and a clear decrease of HDL-C and apoA1 was observed from quartile 1 to quartile 4 (***Table 2***). In the same way, the lipid and lipoprotein levels in the normal weight group were analyzed and the same results were found, as seen in ***Table 3***. However, the corresponding parameters' levels in every quartile were higher in the overweight group than those in the normal weight group except HDL-C level.

**Table 2:** The occurrence of CHD and lipid and lipoprotein levels divided into quartiles of apoB/apoA1 ratio in the overweight group

Characteristic	Qurtiles of apoB/apoAl ratio				*P*^a^
	Quartile 1 (*n*=100)	Quarrtile 2 (*n*=102)	Quarrtile 3 (*n*=99)	Quarrtile 4 (*n*=99)	
CHD [*n*(%)]	66(66)	72(71)	74(75)	88(89)	< 0.001
TG (mmol/L)	1.55 ± 0.81	1.92 ± 1.15	2.04 ± 1.44	2.21 ± 1.19	< 0.001
TC (mmol/L)	3.53 ± 0.83	4.08 ± 0.74	4.41 ± 1.06	5.02 ± 0.97	< 0.001
LDL-C(mmol/L)	1.94 ± 0.54	2.43 ± 0.52	2.68 ± 0.61	3.21 ± 0.72	< 0.001
HDL-C(mmol/L)	1.18 ± 0.33	1.12 ± 0.24	1.05 ± 0.23	1.03 ± 0.20	< 0.001
ApoB (mg/L)	0.59 ± 0.15	0.78 ± 0.12	0.89 ± 0.14	1.12 ± 0.21	< 0.001
ApoA1 (mg/L)	1.15 ± 0.23	1.06 ± 0.17	0.99 ± 0.16	0.96 ± 0.16	< 0.001
ApoB/ ApoA1	0.51 ± 0.09	0.73 ± 0.06	0.90 ± 0.07	1.17 ± 0.20	< 0.001

^a^*P*-Value refers to difference between quartiles 1 and 4. CHD: coronary heart disease; TC: total cholesterol; TG: triglyceride; LDL-C: low density lipoprotein cholesterol; HDL-C: high density lipoprotein cholesterol.

**Table 3:** The occurrence of CHD and lipid and lipoprotein levels divided into quartiles of apoB/apoA1 ratio in the normal weight group

Characteristic	Qurtiles of apoB/apoAl ratio				*P*^a^
	Quartile 1 (*n*=122)	Quarrtile 2 (*n*=113)	Quarrtile 3 (*n*=86)	Quarrtile 4 (*n*=76)	
CHD [*n*(%)]	59(48)	58(51)	47(55)	53(70)	< 0.001
TG (mmol/L)	1.21 ± 0.63	1.36 ± 0.99	1.56 ± 0.70	1.77 ± 0.95	< 0.001
TC (mmol/L)	3.64 ± 0.76	4.00 ± 0.85	4.33 ± 0.87	4.66 ± 1.25	< 0.001
LDL-C(mmol/L)	1.98 ± 0.53	2.26 ± 0.52	2.64 ± 0.57	2.92 ± 0.11	< 0.001
HDL-C(mmol/L)	1.32 ± 0.32	1.28 ± 0.33	1.12 ± 0.23	1.02 ± 0.23	< 0.001
ApoB (mg/L)	0.59 ± 0.12	0.74 ± 0.13	0.87 ± 0.14	1.06 ± 0.26	< 0.001
ApoA1 (mg/L)	1.19 ± 0.23	1.10 ± 0.18	1.03 ± 0.16	0.93 ± 0.18	< 0.001
ApoB/ ApoA1	0.50 ± 0.08	0.68 ± 0.05	0.84 ± 0.05	1.13 ± 0.20	< 0.001

^a^*P* refers to difference between quartiles 1 and 4. CHD: coronary heart disease; TC: total cholesterol; TG: triglyceride; LDL-C:low density lipoprotein cholesterol; HDL-C: high density lipoprotein cholesterol; apoB apolipoprotein B; apoA1 apolipoproteinA1.

**Fig. 1:**
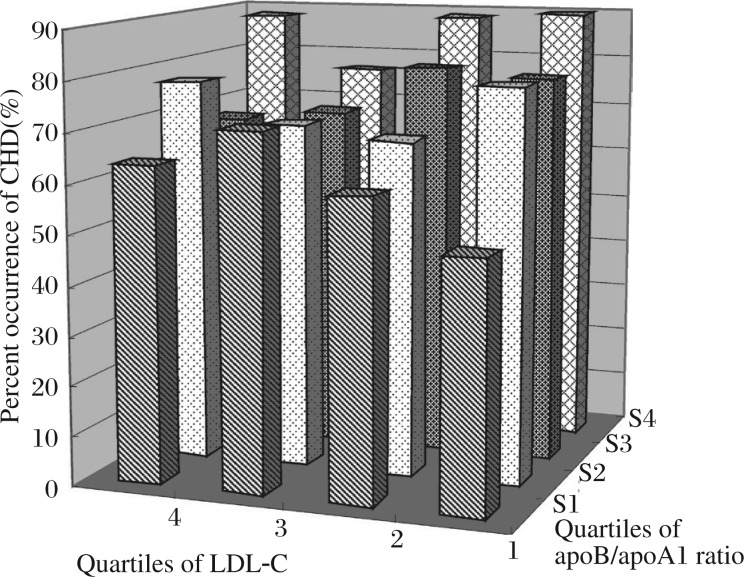
Percentage of CHD occurrence in the over-weight group by quartiles of apoB/apoA1 and LDL-C. CHD: coronary heart disease; LDL-C: low density lipoprotein cholesterol; apolipoprotein B: apoB; apolipoproteinA1: apoA1.

### Percentage of CHD occurrence in the over-weight and obesity group by quartiles of apoB/A1 and LDL-C

***Fig. 1*** shows the percentage of CHD occurrence in the overweight group by quartiles of apoB/apoA1 ratio and LDL-C concentrations. In the highest LDL-C quartile, the percentage of CHD was not always the highest, such as the percentage of CHD in quartile 4 of LDL-C and quartile 1 of apoB/apoA1 ratio was lower than that in quartile 1 of LDL-C and quartile 4 of apoB/apoA1 ratio, and in quartile 1 of LDL-C, and the percentage of CHD in quartile 4 of apoB/apoA1 ratio was almost twice greater than that in quartile 1 of apoB/apoA1 ratio. But in the highest apoB/apoA1 ratio quartile, the percentage of CHD was higher than that in other quartiles except in tertile 4 of apoB/apoA1 ratio and quartile 3 of LDL-C. So in our opinion, apoB/apoA1 is a better CHD risk marker than LDL-C in overweight subjects. This ratio integrates the risk associated with an imbalance between the atherogenic and anti-atherogenic lipoproteins into one number.

### The apoB/apoA1 ratio in the overweight group at baseline was correlated with cardiovascular events

In the overweight and normal weight groups, a multiple logistic regression analysis of CHD events as a dependent variable and all variables listed in ***Table 1*** as explanatory variables was performed, respectively (***Table 4***). As seen in ***Table 4***, age, ever smoking, HDL-C and apoB/apoA1 turned out to be of significant importance for cardiovascular events in the overweight group. But age, glucose glucose (GLU), HDL-C and LDL-C turned out to be of significant importance for cardiovascular events in the normal weight group.

**Table 4:** Logistic regression in the normal weight and overweight group

	B	SEM	Wald	df	*P*	Exp(B)	95%Confidence Interval for Exp(B)	
							Lower	Upper
Over-weight								
Age	0.550	0.190	0.070	1	0.004	1.057	1.017	1.097
Ever smoked	0.911	0.362	5.005	1	0.025	2.250	1.106	4.578
HDL-C	-2.803	0.843	11.051	1	0.001	0.061	0.012	0.317
ApoB/apoA1	1.637	0.760	4.628	1	0.031	5.129	1.156	22.749
Normal-weight								
Age	0.107	0.014	60.100	1	0.000	1.113	1.083	1.143
GLU	0.182	0.060	9.117	1	0.003	1.199	1.066	1.350
HDL-C	-2.704	0.494	29.999	1	0.000	0.067	0.025	0.176
LDL-C	0.387	0.190	4.131	1	0.042	1.472	1.014	2.138

HDL-C: high density lipoprotein cholesterol; apoB: apolipoprotein B; apoA1: apolipoproteinA1; GLU:glucose; LDL-C:low density lipoprotein cholesterol. B: Regression coefficient.

ROC curve analysis of the overweight subjects with or without CHD was performed for apoB/apoA1 and other lipid biomarkers like TC, TG, HDL-C, LDL-C, apoB, and apoA1 (***Table 5***). Area under ROC curve (AUC) was the highest for apoB/apoA1 (0.655). The cut-off point of apoB/apoA1 for optimal sensitivity and specificity was 0.80 with sensitivity at 57.19% and specificity at 71.72%. Comparable ROC curves of apoB/apoA1 were obtained in men (AUC: 0.634, optimal cut-off point: 0.918) and women (AUC: 0.659, optimal cut-off point: 0.805). With the proposed cut-off point of apoB/apoA1, disagreement between direct coronary angiography and apoB/apoA1 was observed in 29 cases (14%) of the overweight subjects. For normal weight subjects, the same method was used and AUC was found to be the highest for TG (0.732), and AUC of apoB/apoA1 was 0.426 (*P* = 0.012). Therefore, we believed the effects of lipids and lipoprotein on the normal weight and overweight groups were different, and apoB/apoA1 on the incidence of coronary heart disease among overweight subjects was a more meaningful prediction.

**Table 5:** Area under the curve for lipid and lipoprotein in over-weight subjects

Test Result Variable (s)	Area	SEM^a^	Asymptotic Sig^b^	Asymptotic 95% Confidence Interval	
				Lower Bound	Upper Bound
TG	0.597	0.033	0.004	0.531	0.662
TC	0.480	0.033	0.555	0.415	0.545
HDL-C	0.295	0.030	0.000	0.237	0.353
LDL-C	0.519	0.033	0.566	0.455	0.583
ApoA1	0.267	0.028	0.000	0.213	0.322
ApoB	0.521	0.033	0.524	0.458	0.585
ApoB/ApoA1	0.655	0.029	0.000	0.598	0.712

The test result variable (s): TG, TC, HDL, LDL, apoA, apoB, and apoB/ apoA1 had at least one tie between the positive actual state group and the negative actual state group. Statistics may be biased. ^a^Under the nonparametric assumption; ^b^Null hypothesis: true area = 0.5. TG: triglyceride; TC: total cholesterol; HDL-C: high density lipoprotein cholesterol cholesterol; LDL-C: low density lipoprotein cholesterol; apoB: apolipoprotein B; apoA1: apolipoproteinA1.

### The correlation coefficients between the apoB/apoA1 ratio and individual components in the overweight group

The correlation coefficients between the apoB/apoA1 ratio and individual components in the overweight group are shown in ***Table 6***. There was a statistically positive correlation (*P* < 0.05) between the apoB/apoA1 ratio and BMI, WHR, GLU, TG, TC, and LDL-C, but a significantly negative correlation between the apoB/apoA1 ratio and HDL-C (*P* < 0.05). The strongest correlation was found between the apoB/apoA1 ratio and LDL-C (*r* = 0.626), and the second strongest was between the apoB/apoA1 ratio and TC (*r* = 0.531). Furthermore, apoB/apoA1 ratio and hs-CRP, and WBC had a statistically positive correlation.

**Table 6:** The correlations between the apoB/apoA1 ratio and individual components in the over-weight group

	BMI	WHR	GLU	TG	TC	HDL-C	LDL-C	Hs-CRP	WBC
*r*	0.119	0.196	0.124	0.305	0.531	-0.248	0.626	0.242	0.202
*P*	0.017	< 0.001	0.013	< 0.001	< 0.001	< 0.001	< 0.001	< 0.001	< 0.001

BMI: body mass indexs; WHR: waist hip ratio;GLU:Glucose; TG: triglyceride; TC: total cholesterol; HDL-C: high density lipoprotein cholesterol; LDL-C: low density lipoprotein cholesterol; Hs-CRP: highsensitivity C-reactive protein; WBC: white blood cell.

## DISCUSSION

Cardiovascular (CV) disease is the most common cause of death in the world. The identification of individuals at increased CV risk represents a priority. Data from the present study showed that populations who have higher BMI, larger waist-hip-ratio, and higher mean values of SBP and DBP, and also higher levels of serum triglycerides and cholesterol were more likely to suffer a cardiovascular event. Actually, LDL-C, closely related to CV disease and mortality, remains the cornerstone of lipid management[13]. The development of refined lipoprotein assessment helped our understanding of the atherosclerotic process. So many reports attribute more weight to the apoB/apoA1 ratio as an index of CV risk[14]. As to the determination of LDL-C alone, the present study demonstrates that the apoB/apoA1 ratio is an identified effective CV risk factor in overweight CHD subjects, and LDL-C alone is insufficient[15]. The combined detection of LDL-C and the apoB/apoA-I ratio proved very effective in pinpointing subjects amenable to prevention even when apparently “protected” by normal cholesterol values.

ApoB is the major apolipoprotein in all potentially atherogenic lipoprotein particles[16], i.e. VLDL, IDL, and LDL particles. There is only one apoB per particle. Thus, plasma value of the total apoB reflects the number of cholesterol and, to some degree, triglyceride containing particles. The most abundant apoB particle is the small dense LDL, which constitutes about 90% of the whole apoB population. SdLDL is also considered to be more atherogenic than the large buoyant LDL particles[17]. So, not all LDL particles are the same[18]. A total LDL-C value cannot distinguish between the less harmful, so called large buoyant LDL, and the more atherogenic sdLDL particles. ApoA1 is the major apolipoprotein in the HDL particles. ApoA1 is a main initiator and “driver of the reverse cholesterol transport”. ApoA1 can also manifest anti-oxidant and anti-inflammatory effects[19], so it can stimulate both endothelial production of nitric oxide as well as release of prostacyclin from the endothelium. Thus, apoA1 manifests several anti-atherogenic effects. Therefore, the apoB/apoA1 ratio reflects the balance between pro-atherogenic IDL, VLDL, LDL particles and anti-atherogenic HDL particles. In AMORIS (the biggest prospective study, Apolipoprotein-related MOrtality RISk study)[20], the apoB/apoA1 ratio was the single best lipid-related risk variable, also considering the other conventional lipids and lipid ratios. The higher the level of the apoB/apoA1 ratio is, the more likely cholesterol is to be deposited in the arterial wall, thereby provoking atherogenesis[21]. In our study, the comparison between CHD patients and healthy subjects was performed and TG and apoB/apoA1 ratio levels were found higher and HDL-C and apoA1 levels in CHD patients were lower. No significant differences between the two groups in TC, and LDL-C and apoB were observed. Therefore, we believe that apoB/apoA1 levels were better than LDL-C levels in predicting the risk of CHD.

Obesity is a cause of CHD risk factors[22],[23]. The overall hazard ratios for all-cause and CVD mortality in persons with the metabolic syndrome(MetS) as compared with persons without it were 1.44 and 2.26 in men and 1.38 and 2.78 in women after adjustment for age, blood cholesterol levels, and smoking[24],[25]. Epidemiological investigations revealed that the occurrence of CHD in the obese population was 2.0-2.5 times that in people with a normal BMI[26]. So according to BMI, the subjects were divided into two groups, and the lipids and lipoproteins of the normal and overweight groups were observed. High mean levels of TC, TG, LDL-C and apoB and low HDL-C and apoA1 characterized the overweight group. We found that the overweight group with increased levels of apoB/apoA1 ratio had an increased risk for CHD, compared to groups with lower levels. The highest quartile of apoB/apoA1 in this sample corresponded to a clear increase in the occurrence of CHD. But in the normal weight group, the highest quartile of apoB/apoA1 did not correspond to a clear increase in the occurrence of CDH. Furthermore, the area under the curve was observed, and the apoB/apoA1 ratio area under the curve was the largest in the overweight group, while TG was the largest in the normal weight groups. Therefore, we believed that apoB/apoA1 had different significances for different populations, and it was more important for the overweight group to predict the occurrence of CHD. Maybe, part of the reason is that obesity was a “conditional” risk for developing CV risk factors such as diabetes, dyslipidemia, hypertension, and obstructive sleep apnoea[27]. The positive correlation between the apoB/apoA1 ratio and hs-CRP and WBC was observed, so we think that apoB/apoA1 ratio was associated with not only lipid, but also with chronic low grade inflammation caused by obesity[28]–[30].

Some study showed people with 27.5 kg/m^2^ ≤ BMI ≤30 kg/m^2^ were at reduced risk of dying[31]. Among patients undergoing percutaneous intervention for coronary artery disease, increased BMI was associated with improved 5-year survival. Among those with established CHD, the adverse effects of excess adipose tissue may be offset by beneficial vasoactive properties. We also analyzed the subjects with 27.5 kg/m^2^ ≤BMI ≤30 kg/m^2^ and BMI > 30 kg/m^2^, and found that there was a higher occurrence of CHD and apoB/apoA1 ratio in people with BMI > 30 kg/m^2^ than that in people with BMI≤30 kg/m^2^. The occurrence of CHD and the apoB/apoA1 ratio had no significant difference between group with 27.5 kg/m^2^ ≤ BMI ≤30 kg/m^2^ and group with BMI < 27.5 kg/m^2^, perhaps because our sample size was not big enough. Except this, we could not deduce the incidence of mortality according to the occurrence of CHD.

In conclusion, apoB and apoA1 were simple clinical indicators, and the apoB/apoA1 ratio was closely related with CHD in the overweight group. The apoB/apoA1 ratio increase indicated an increased risk of CHD and may provide some useful information in the differential diagnosis.

Some limitations have to be taken into consideration. The design of the present study was cross-sectional and the results did not imply causality, so the results had some limitations. So our findings should not be necessarily applicable to the general population.
